# Understanding the lung cancer mortality reductions produced by low-dose CT screening

**DOI:** 10.1016/j.lanepe.2021.100255

**Published:** 2021-11-04

**Authors:** Yuki Furukawa

**Affiliations:** Tokyo Musashino Hospital, Tokyo, Japan; Department of Neuropsychiatry, University of Tokyo Hospital, Tokyo, Japan

I read with great interest the publication entitled “Results from the randomised UKLS trial: Lung cancer mortality reduction by LDCT screening confirmed in an international meta-analysis” [1]. The authors reported the results of the UKLS trial, which was prematurely stopped and therefore underpowered. Results were non-significant both for lung cancer mortality and all-cause mortality in this single trial, but when they were pooled with previous trials, they showed an improvement in lung cancer mortality, as well as in all-cause mortality. They reported the effect sizes, however, only in terms of relative risks, which often lead to readers’ misinterpretations of the magnitude of benefits or harms [2,3]. Providing absolute risks together would be preferable [2,3].

I have calculated the absolute risk differences using numbers provided in the publication [1]. Given the control event rate among people aged 50-75 with an elevated risk of developing lung cancer as found in UKLS trial, which is, in the median follow-up period of 7.3 years, 23 per 1,000 (46/1,981) for lung cancer mortality and 134 per 1,000 (266/1,981) for all-cause mortality, low-dose chest CT screening would lead to 20 per 1,000 [95%CI 18 to 21] for cancer mortality and 130 per 1,000 [126 to 134] for all-cause mortality [1].

I believe that providing this information together with relative risks will make the interpretability of the publication better and enhance the implication of this research for policy making even more.

## Author Contribution

YF conceptualized the letter and wrote it.

## Declaration of Interests

YF declares no conflicts of interest.
